# Sparse attention double-channel FCN network for numerical analysis tracheid features in larch

**DOI:** 10.3389/fpls.2022.1079556

**Published:** 2022-12-22

**Authors:** Chao Li, Lixin Zhang, Saipeng Wang, Xun Chen, Weipeng Jing

**Affiliations:** College of Information and Computer Engineering, Northeast Forestry University, Harbin, China

**Keywords:** larch, tracheid features, FCN, dual channel, sparse attention, feature selection

## Abstract

Understanding the macro-mechanical behavior of wood at the micro-scale is of great significance for the design of cell-wall-like composite materials and pulp papermaking. In order to predict tracheid mechanical properties and analyze its relationship with tracheid features, based on the FCN network model, a double-channel FCN network with sparse attention (D-SA-FCN) was designed by introducing the double-channel mechanism and the sparse attention mechanism. The features of tracheid of larch were extracted numerically and the data set was established by using the compression strength data, the gray level co-occurrence matrix, cell segmentation and geometric analysis. A feature analysis algorithm based on PCA and random forest was established to optimize the feature values. The training set accuracy of the D-SA-FCN network model reached 85.75% with the five-level mechanical property level according to the classification standard. The accuracy of the training model is 71.48% and 79.52% when the morphological and texture features are input respectively. The results show that texture features had a more significant impact on mechanics to a certain extent and the D-SA-FCN could reduce the computational complexity and improve the prediction accuracy.

## 1 Introduction

Wood is a kind of natural raw material, with green, easy processing, renewable and other performance features, in production and life and other aspects occupy an important position. As a structural material, wood has elasticity and toughness, good seismic and impact resistance, small thermal conductivity, belongs to the category of thermal insulation materials, in a dry environment for a long time, is not easy to conduct, and has good durability; As a building material, wood has a small dead weight and high bearing capacity, which is renewable, degradable and easy to construct. Based on the above advantages, wood is widely used in industry, agriculture and daily life and other fields.

Many scholars have long been engaged in the treatment and analysis of wood micro-structure. IIic (1983) developed a wood cell image processing system based on the basic knowledge of image processing, which can obtain many features of wood microscopic images and extract the area ratio and cell cavity area from the cell xylem. Donaldson (1998) used digital image analysis technology to measure the size of wood cells and com-pared the differences between images collected by conventional transmission light microscopy and confocal laser scanning electron microscopy as digital image sources. Ning et al. (2005) used image analysis based on digital image processing technology to achieve digital measurement of wood tissue ratio, saturation rate, control distribution density, cell wall thickness, diameter/chordal diameter, wall cavity ratio and morphological amount. Mohan et al. (2014) extracted wood microscopic image features through grey co-occurrence matrix and other methods. MITOBABA (2017) summarizes the calculation methods of DOL coefficient or DOL adjustment coefficient obtained by DOL prediction model in timber structure design codes. Kinjouo et al. (2021) analyzed of the variability of xylem anatomical features were done by semi-automatic measurements using the SpectrumSee digital image analysis software. Kitin et al. (2021) distinct chemical fingerprints of the wood of Afzelia pachyloba and A. bipindensis demonstrated an effective method for identifying these two commercially important species.

The relationship between micro properties and macro properties of wood has been gradually studied by scholars. Figueroa-Mata et al. (2018) obtained the microscopic structure image of wood through an image acquisition device and extracted the visual features of wood by using digital image processing technology, and proposed a wood recognition method with simple operation, high recognition efficiency and low cost. Kvist et al. (2018) used the method of fluorescence recovery after photobleaching (FRAP) to perform diffusion measurements locally in the wood microstructure. Salma et al. (2018) obtained results able to identify the microscopic image of wood as a wood species with average SVM accuracy of 85%. Fahey et al. (2019) designed a method to quickly predict the cell wall composition of solid wood samples through a powerful combination of NIR spectroscopy and PLS regression, thereby avoiding milling. Lens et al. (2020) aimed to review existing computer vision methods and compare the success of species identification based on (1) several image classifiers based on manually adjusted texture features, and (2) a state-of-the-art approach for image classification based on deep learning, more specifically Convolutional Neural Networks (CNNs). Zhao et al. (2021) proposed a classification method of wood species based on the fusion of i-BGLAM texture features and spectral features of hyperspectral images, and the classification accuracy achieved by the fusion method of i-BGLAM extracted texture features and average spectral features can reach 100%. Henriques et al. (2022) updated the elastic modulus parameters for identifying the orthogonal anisotropy of pine by the finite element model. da Silva et al. (2022) introduced a new image dataset containing microscopic images of the three main anatomical sections of 77 Congolese wood species.

Attention mechanism and channel fusion algorithms have gradually attracted the attention of scholars and applied to deep learning algorithms. Zeng et al. (2020) proposed a lightweight neural network based on MobilenetV2, which removed some redundant reverse residual blocks and reduced the channel expansion coefficient of the reverse residual block, greatly reducing the amount of calculation and parameters. Zhu et al. (2021) proposed a wood microscopic image classification method based on decomposition-aggregation network model, which combined image geometric transformation and Mixup data expansion technology. Konovalenko et al. (2021) constructed a classifier based on the integration of two deep residual neural networks ResNet50 network and ResNet152 to detect three types of defects on planar metal surfaces. Fan et al. (2021) proposed a convolutional neural network entity relation classification model integrating location and lexical level feature embedding, and gave the calculation representation method of features. This network entity relation classification model has good classification effect. Yuan et al. (2021) proved that shortening the network length can greatly speed up the image recognition speed, and combining the multi-scale segmentation method can effectively improve the recognition accuracy.

In this paper, the texture features of the wood microscopic image were extracted by gray level co-occurrence matrix. Meanwhile, the morphological features of tracheid were obtained by image segmentation of wood cells. Combined with the random forest algorithm, the analysis model of wood tracheid features and mechanical properties was constructed, and the effects of different properties on its mechanical properties were analyzed.

## 2 Material and data

### 2.1 Material

Larch is the main coniferous tree species in the Greater Hinggan Mountains of China, with abundant wood storage. It is the main forest group species in northeast China and Inner Mongolia, with heavy and solid, strong compressive and bending strength.

Adult larch from Greater Khingan Mountains was selected as the experimental subjects. According to GB/T 1935-2009 (2009) standard, the length, width and height of the sample are 20mm,20mm and 30mm respectively. Random sampling was performed at the junction of the heart sapwood and the normal part of the growth wheel. 200 larch mechanical test specimens were obtained, and then the specimens were numbered one by one.

### 2.2 Data collection

#### 2.2.1 Image data acquisition

**Figure 1:**
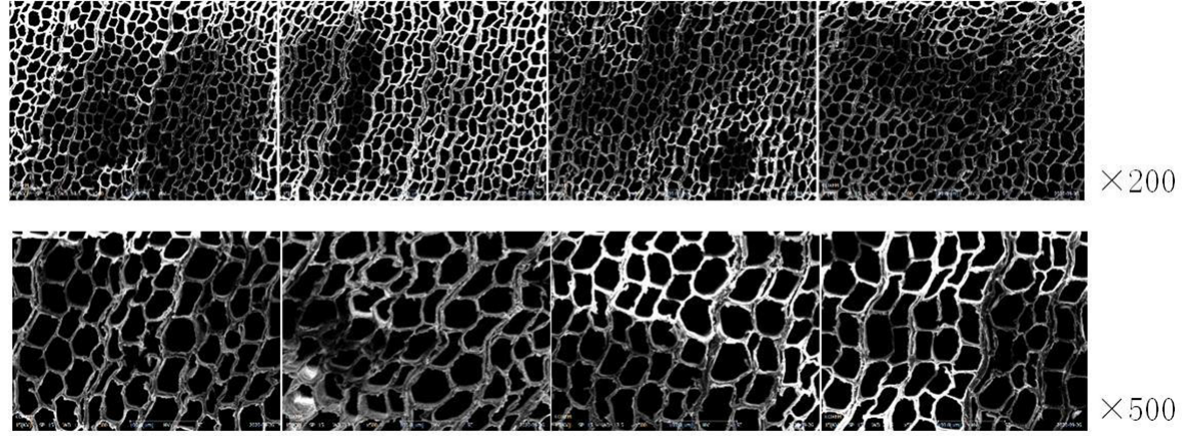
Microscopic images of experimental specimens.

COXEN’s scanning electron microscope (Coxen, made in Korea) was used for image acquisition with a resolution of 1800*1600. The experimental specimen was placed on the loading platform at the bottom of the device, and the surface of the specimen was observed at ×200 and ×500 double magnification by adjusting the focal length of the microscope. The images were captured and saved in the field of vision. At four different positions of the specimen, one image was taken at ×200 and ×500 ratio and eight microscopic images were taken for each specimen. A total of 1600 microscopic images of 1600*1200 size were collected. **Figure 1** shows the microscopic image of specimen No. 1.

#### 2.2.2 Collection of mechanical properties

**Figure 2:**
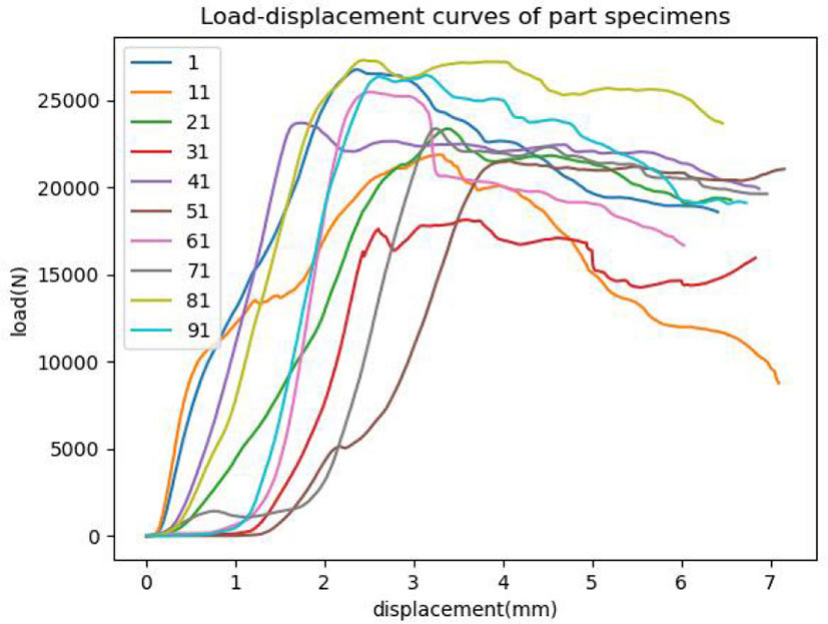
Load variation curve of experimental specimens.

The destructive test of the mechanical properties of larch specimens was carried out by a universal testing machine (Kexin, made in China). The hydraulic cylinder was applied to the larch specimen at a uniform speed, and the specimen was destroyed within 1.5min~2.0min, that is, the load detected by the sensor was significantly reduced. The failure load of the specimen was recorded by the control module of the mechanical property testing device, and a total of 200 groups of failure loads were recorded. **Figure 2** is the load change curve of part specimens.

According to GB/T1935-2009 (2009) when the water content of the specimen is 12%, the compressive strength parallel to the grain of wood of the specimen is calculated according to Formula 1 and is accurate to 0.1mpa.


$$\sigma_{w}=P_{max}/\left(b\cdot t\right)$$

Where, ***σ*_*w*_** is the compressive strength parallel to the grain of wood when the moisture content of the specimen is 12% (MPA); *P*_*max*_ is failure load (N); ***b*** is sample width (mm); ***t*** is thickness of sample (mm).

According to the calculation formula of the failure load and the compressive strength, the compressive strength parallel to the grain of wood of the test piece was calculated, and the grade of larch was divided according to the performance classification standard of Larch (GB50005-2017, 2017), which was used as the basis of model classification. The classification results are shown in **Table 1**.

**Table 1:** Standard for classification of larch properties.

Tree species	Material grade	*σ*_*w*_ (N/mm2)	number ofsamples
Larch	Ic	>22.5	92
	IIc	>18.9	54
	IIIc	>16.9	17
	IVc	>14.0	8
	Vc	other	29

## 3 Method

### 3.1 Method flow

**Figure 3:**
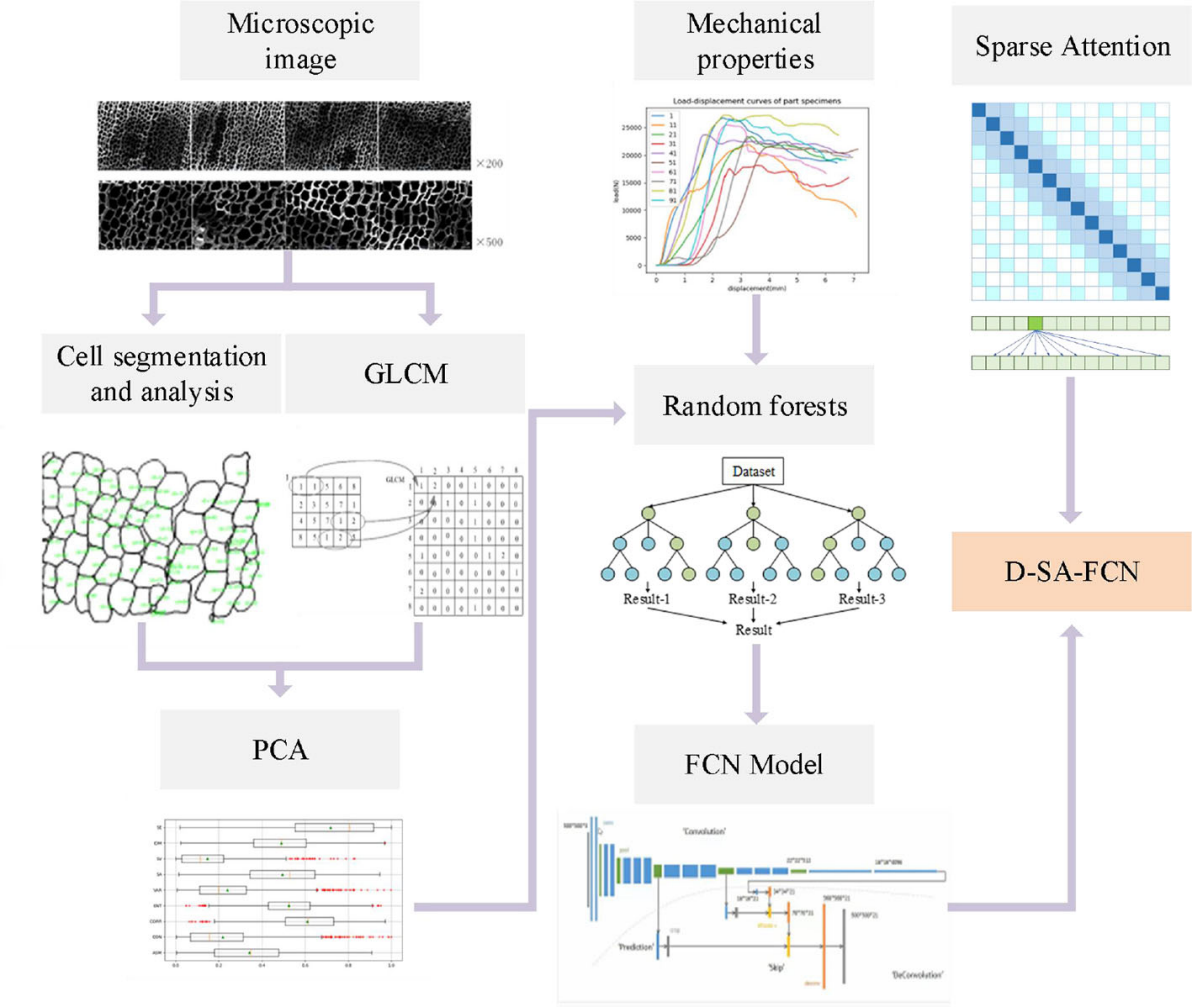
Method flow chart.

As shown in **Figure 3**, after the mechanical data and microscopic image data were collected and pre-processed, the eigenvalues of the grey co-occurrence matrix in the microscopic image were extracted as the tracheid texture eigenvalues, and the average tracheid area and average tracheid circumference were extracted as the morphological eigenvalues. Combined with numerical analysis, a random forest model was used to optimize the eigenvalues. With the optimized features and processed microscopic images as the input of the network, and mechanical properties as the classification basis, a D-SA-FCN neural network model was established. The innovation and usability of the model were determined by comparing various classification models. Meanwhile, the sparse attention mechanism is introduced to construct the D-SA-FCN network model. According to the model classification results, combined with the input parameters to complete the numerical analysis.

### 3.2 Extraction of features

In this paper, the grey co-occurrence matrix was used to extract the texture features of the tracheid microscopic image. The grey co-occurrence matrix described the texture features of the image by calculating the spatial correlation features of grey. The eigenvalues were the grey distribution information of the image in the direction, local neighborhood and variation amplitude.

The difference in position relation also can cause the difference in the grey level co-occurrence matrix. The commonly used position relation is 0°, 45°, 90° and 135°. To improve the operation efficiency, the position relation of 0° was taken in this paper. Through comparative analysis of many tests, the sliding window size is 8*8, the grey level was 16, and the step size was 4. Nine texture features were extracted by using a grey co-occurrence matrix, which was Angle second-order distance, contrast, correlation, entropy, variance, mean sum, variance sum, deficit moment, and entropy. The specific calculation formula is as **Table 2**.

**Table 2:** The specific calculation formula of texture features.

Features	Formula
Angular Second Moment (ASM)	$W_{1}=\sum_{i=1}^{g}\sum_{j=1}^{g}m^{2}\left(i,j\right)$
Contrast (CON)	$W_{2}=\sum_{i=1}^{g}\sum_{j=1}^{g}{\left(i-j\right)}^{2}•m\left(i,j\right)$
Correlation (CORR)	$W_{3}=\sum_{i=1}^{g}\sum_{j=1}^{g}\left[\left(ij\right)•m\left(i,j\right)-u_{i}u_{j}\right]/\delta_{i}\delta_{j}$
Entropy (ENT)	$W_{4}=-\sum_{i=1}^{g}\sum_{j=1}^{g}m\left(i,j\right)•logm\left(i,j\right)$
Variance (VAR)	$W_{5}=-\sum_{i=1}^{g}\sum_{j=1}^{g}{\left(i-a\right)}^{2}•m\left(i,j\right)$
Sum of Average (SA)	$W_{6}=\sum_{k=2}^{2g}k•\sum_{i=1}^{g}\sum_{j=1}^{g}m\left(i,j\right)\;k=2,3,\ldots ,2g$
Sum of Variance (SV)	$W_{7}=\sum_{k=2}^{2g}\left(k-W_{6}\right)•\sum_{i=1}^{g}\sum_{j=1}^{g}m\left(i,j\right)$
Inverse Difference Moment (IDM)	$W_{8}=\sum_{i=1}^{g}\sum_{j=1}^{g}m\left(i,j\right)/\left[1+{\left(i-j\right)}^{2}\right]$
Sum of Entropy (SE)	$W_{9}=-\sum_{k=2}^{2g}P_{x}\left(k\right)•logP_{x}\left(k\right)$

Ps: Where, **m**(**i**, **j**) is the grey value at coordinate (**i**, **j**) ; u_**i**_ and u_**j**_ are the mean value of grey value in the direction of **i** and **j** respectively; δ_**i**_ and δ_**j**_ are the standard deviation of grey value in the direction of **i** and **j** respectively.

The extracted eigenvalues of the grey co-occurrence matrix of some images are shown in **Table 3**.

**Table 3:** Eigenvalues of the grey co-occurrence matrix.

Sample ID	ASM	CON	CORR	ENT	VAR	SA	SV	IDM	SE
1-1	0.5271	0.6098	0.5259	0.4820	0.4072	0.6850	0.2717	0.4500	0.9468
20-1	0.2250	0.2171	0.7494	0.6848	0.3281	0.4007	0.0458	0.3521	0.6363
40-1	0.3396	0.1762	0.3460	0.4920	0.0931	0.5222	0.1095	0.3967	0.7968
60-1	0.3459	0.1176	0.6248	0.5158	0.1305	0.5283	0.1138	0.4717	0.8039
80-1	0.1405	0.1467	0.7780	0.6869	0.5972	0.2917	0.0160	0.3745	0.4678
100-1	0.4423	0.1466	0.6925	0.5031	0.1829	0.6155	0.1895	0.4939	0.8933
120-1	0.2093	0.6515	0.4631	0.7502	0.4667	0.3820	0.0392	0.1623	0.6087
140-1	0.2804	0.0812	0.7467	0.5314	0.1434	0.4623	0.0733	0.5334	0.7220
160-1	0.4008	0.1768	0.6475	0.5026	0.1692	0.5791	0.1545	0.4926	0.8588
180-1	0.0457	0.0869	0.7232	0.7289	0.2217	0.1265	0.0000	0.3919	0.1948
200-1	0.1365	0.0618	0.9110	0.6254	0.2811	0.2859	0.0150	0.6038	0.4585

### 3.3 Morphological features

**Figure 4:**
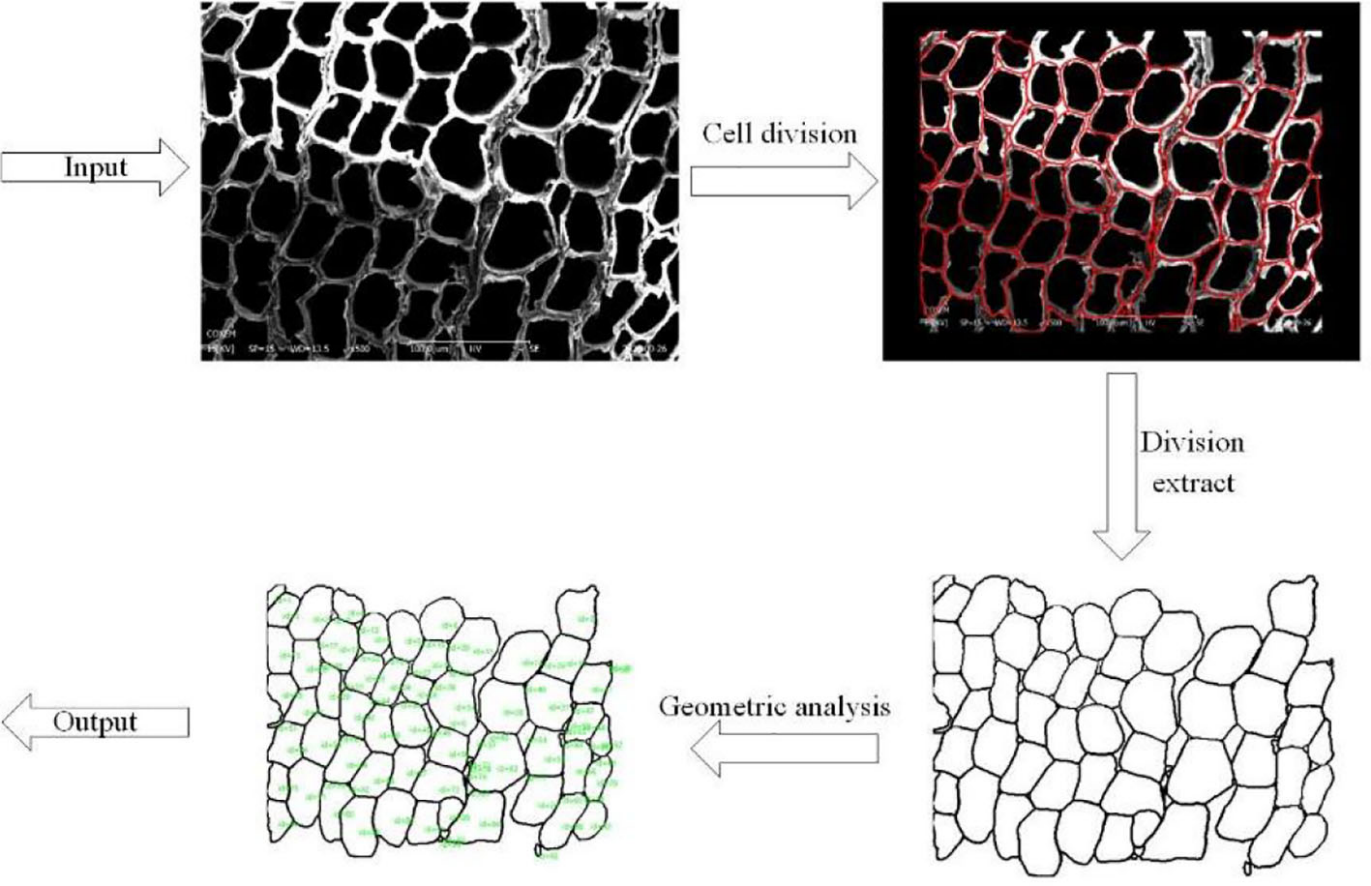
Cell segmentation and morphological analysis.

The morphology of the tracheid affects the physical properties of wood. It is helpful to judge the mechanical properties of wood by analyzing tracheid size, wall cavity ratio, length to width ratio and other tracheid properties. The premise of morphological feature extraction of the tracheid is tracheid segmentation. The manual labeling method is time-consuming and labor-consuming and subject to subjective factors. Although the watershed algorithm can improve efficiency, it will ignore the tracheid wall to some extent. In this paper, a universal cell segmentation method based on deep learning, was used to complete the segmentation of tracheid in microscopic images. On this basis, labeled area analysis was used to calculate the average tracheid area and average tracheid perimeter in microscopic images. **Figure 4** shows the extraction process of the tracheid.

For two kinds of images with 200 and 500 magnification, different scales are used for conversion. The morphological features of microscopic images such as aver-age tracheid area (AREA) and average tracheid perimeter (PERIMETER) will be extracted, as shown in **Table 4**.

**Table 4:** Features of tracheid morphology.

Sample ID	AREA(µ*m* ^2^)	PERIMETER(µ*m*)		Sample ID	AREA(µ*m* ^2^)	PERIMETER(µ*m*)
1-1	602.0309	98.3547		200-1	742.7495	114.8171
1-2	635.3317	101.6269		200-2	630.2454	110.5148
1-3	596.1804	100.2496	⁝	200-3	713.6520	111.8941
1-4	591.0365	99.3771		200-4	672.1085	108.6747
1-5	549.4071	95.7480		200-5	603.5202	101.4500
1-6	563.5833	99.5033		200-6	658.5693	107.1606
1-7	614.2355	99.2346		200-7	626.2355	109.2633
1-8	574.2416	96.1345		200-8	637.9235	110.5322

### 3.4 Numerical analysis

**Figure 5:**
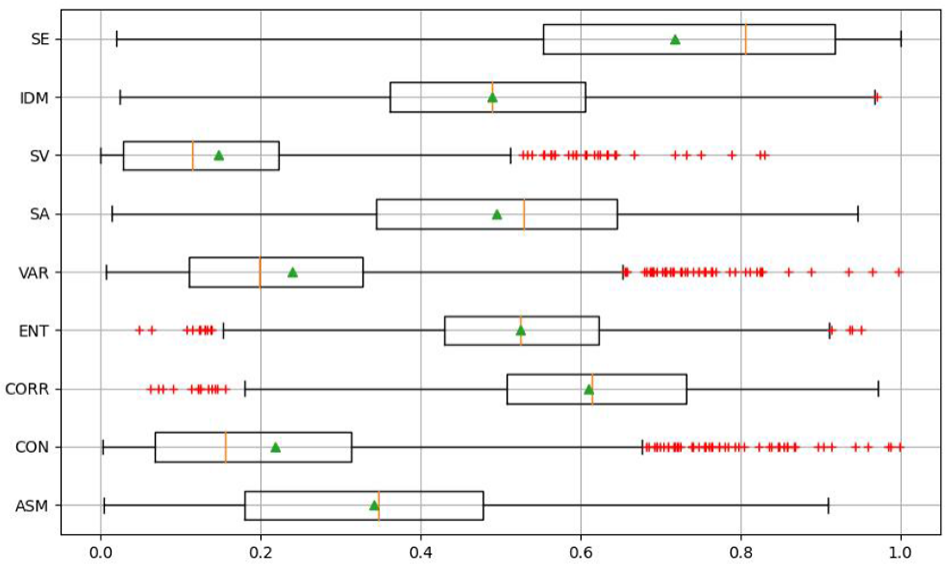
Texture feature outliers removed.

Although random forest can complete feature optimization, if the input redundancy is too high, the effectiveness of optimization will be affected. Therefore, before feature optimization through the random forest model, the redundancy between feature variables should be reduced. In this paper, by calculating the correlation coefficient between the parameters, the redundant parameters are analyzed and screened out. The correlation coefficient matrix is shown in the **Table 5**. And as can be seen from it, the correlation between ASM and SA, SV and SE are 0.986, 0.955 and 0.935, respectively. ASM with more independent parameters is selected. Finally, the optimal texture features are ASM, CON, CORR, ENT, VAR and IDM. As shown in **Figure 5**, abnormal data were eliminated according to the boxplot of each texture feature. According to standard 3-segama, 43 pieces of data were removed, resulting in 1557 pieces of remaining data.

**Table 5:** Correlation coefficients among features.

	ASM	CON	CORR	ENT	VAR	SA	SV	IDM	SE
ASM	1.000	0.170	0.209	0.330	0.147	0.986	0.955	0.421	0.935
CON	0.170	1.000	0.304	0.334	0.411	0.220	0.053	0.607	0.282
CORR	0.209	0.304	1.000	0.171	0.267	0.236	0.139	0.263	0.265
ENT	0.330	0.334	0.171	1.000	0.569	0.429	0.505	0.357	0.690
VAR	0.147	0.411	0.267	0.569	1.000	0.139	0.176	0.541	0.111
SA	0.986	0.220	0.236	0.429	0.139	1.000	0.896	0.408	0.980
SV	0.955	0.053	0.139	0.505	0.176	0.896	1.000	0.445	0.789
IDM	0.421	0.607	0.263	0.357	0.541	0.408	0.445	1.000	0.361
SE	0.935	0.282	0.265	0.690	0.111	0.980	0.789	0.361	1.000

### 3.5 Feature selection

The random forest model not only has been widely used in the classification problem but also has a certain application in feature selection, this is because the random forest model in fitting data, the input parameters have a measure of the importance of a variable, the numerical random forest model after fitting thought given to the importance of the in-put parameters. The larger the value of the variable importance measure is, the more important the corresponding input parameter is for the accuracy of classification.

#### 3.5.1 Decision tree

The decision tree is a basic classifier that divides features into two categories. The constructed decision tree has a tree-shaped structure and can be considered as a collection of IF-THEN rules. The main advantages of the model are readability and fast classification speed.

The influencing parameters of decision tree classification ability are as follows: maximum number of features to be selected, maximum depth of decision tree, the mini-mum number of samples required for internal node redivision, the minimum number of samples of the leaf node, minimum sample weight of leaf node, the maximum number of the leaf node, and minimum impurity of node division.

#### 3.5.2 Random forest and its indicators

A decision tree is constructed by using sub-datasets and randomly selected features. All decision trees constitute a random forest, and each decision tree outputs a result. By voting the judgment results of the decision tree, the output result of the random forest is obtained.

There are two factors influencing the accuracy of random forest classification: first, the correlation between any two trees in the forest, the greater the correlation, the lower the accuracy; Second, the classification ability of each tree in the forest, the stronger the classification ability of each tree, the higher the accuracy of the whole forest.

#### 3.5.3 Establishment of random forest network model

The data set was composed of 1557 specimens. In each data, angular second moment, contrast, entropy, negative moment, correlation, average tracheid area, and average tracheid circumference were used as the classification features of random forest, and the compressive strength parallel to the grain of wood was used as the actual features. The model sets the number of training sets as 1401, and the number of test sets as 156.

A different number of the decision tree model accuracy, and different maximum depth model accuracy, when the number of decision trees to 900, and when the maximum depth of 35, the classification accuracy of 72.25%, and increase the number of decision trees or maximum depth, accuracy will not be promoted and even fell. The training accuracy rate of the test set was 72.25%, and the average absolute error was 5.57.

**Figure 6:**
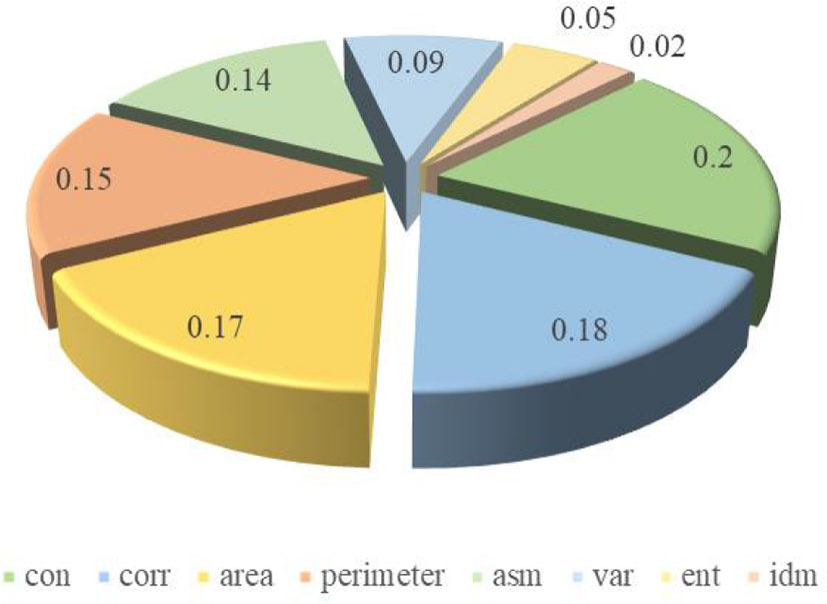
The weighted value of each input parameter.

According to the random forest model, the influence degree of each input parameter on the results is shown in **Figure 6**, CON, CORR, AREA, PERIMETER and ASM have a greater impact, accounting for 20%, 18%, 17%, 15% and 14%. Among them, VAR, ENT and IDM have less influence, accounting for 9%, 5% and 2%. Finally, CON, CORR, AREA, PERIMETER and ASM were selected as the selected feature parameter arrays, and they were input into the random forest network model again, and the accuracy reached 75.31%.

### 3.6 Sparse attention

The attention mechanism in neural networks is a resource allocation scheme to allocate computing resources to more important tasks and solve the problem of information overload when computing capacity is limited. This paper adopts a novel self-attention mechanism called the ed sparse attention mechanism. The main purpose of the sparse attention mechanism is to reduce traditional Transformer’s time and space complexity. With a top-k selection, attention is reduced to sparse attention, the most helpful part of the attention is retained, and other irrelevant information is removed. This selective approach is effective in preserving important information and eliminating noise. Attention can be focused more on the value factors that contribute the most.

In the self-attention layer, the input embedding matrix *X* is mapped to the output matrix and parameterized by the connection mode *S*={*S*_1_,⋯,*S*_*n*_} , where *S*_*i*_ represents the index set of the input vector corresponding to the *i* th output vector. The output vector is a weighted sum of the transformations of the input vectors.


$$\text{Attend}\left(X,S\right)={\left(a\left(x_{i},S_{i}\right)\right)}_{i\in \left\{1,\cdots ,n\right\}}$$

$$a\left(x_{i},S_{i}\right)=\text{softmax}\left(\frac{\left(W_{q}x_{i}\right)K_{S_{i}}^{T}}{\sqrt{d}}\right)V_{S_{i}}$$

$$K_{S_{i}}={\left(W_{k}x_{j}\right)}_{j\in S_{i}}\;\;\;V_{S_{i}}={\left(W_{v}x_{j}\right)}_{j\in S_{i}}$$

Where, *W*_*q*_ , *W*_*k*_ , and *W*_*v*_ represent the weight matrices which transform a given *x*_*i*_ into a query, key, or value, and *d* is the inner dimension of the queries and keys. The output at each position is a sum of the values weighted by the scaled dot-product similarity of the keys and queries.

#### 3.6.1 Attention comparison

**Figure 7:**
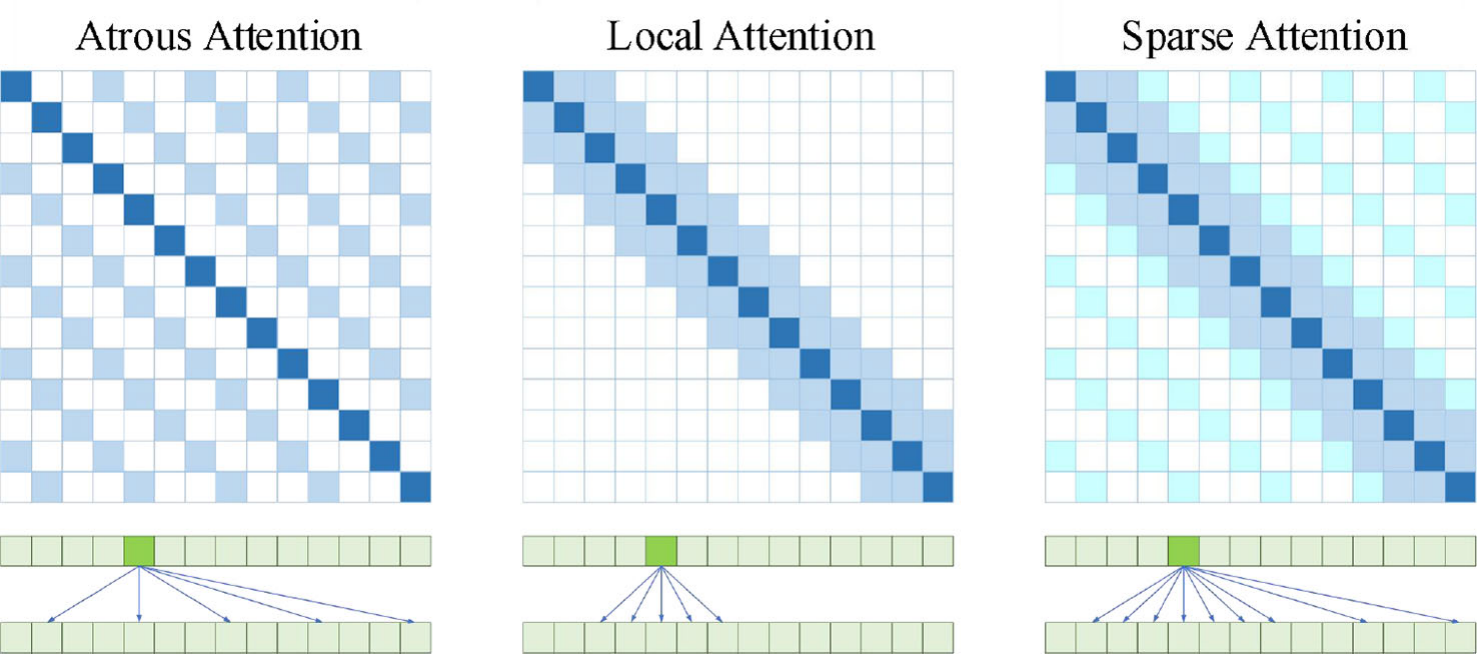
Attention matrix and correlation diagram of three kinds of Self Attention.

As shown in **Figure 7**, Atrous Attention imposes a constraint on the correlation by forcing each element to be only at a distance of *k*,2*k*,3*k*,⋯ . Where *k*>1 is a preset hyperparameter. Since computational attention is now “hopping”, each element is actually only related to about *n*/*k* elements, so that the efficiency and memory usage are ideally *O*(*n* ^2^/*k*) . In other words, it can go straight down to the original 1/*k* . Local Attention gives up global association and introduces local association. Each element is only associated with *k* elements before and after it and itself, that is, a window of 2*k*+1 size, whose time complexity is *O*((2*k*+1)*n) , that is, it grows linearly with n, but sacrifices long-distance correlation. Sparse Attention combining the first two. The attention value is set to 0 for all locations except those with a relative distance of no more than *k* and a relative distance of 2*k*,3*k*,⋯ . Local tight correlation and remote sparse correlation.

#### 3.6.2 Sparse attention combination

Standard intensive attention is simply a linear transformation of defined participating functions.


$$\text{attention}\left(X\right)=W_{p}•\text{attend}\left(X,S\right)$$

Where, *W*_*p*_ denotes the post-attention weight matrix.

The first way is to use an attention type for each remaining block and interlace them either sequentially or in a proportion determined by the hyperparameters.


$$\text{attention}\left(X\right)=W_{p}•\text{attend}\left(X,A^{\left(r\;\text{mod}\;p\right)}\right)$$

Where, *r* is the index of the current residual block and *p* is the number of factorized attention heads.

Another way is to use multi-head attention, where n attention products are computed in parallel and then concatenated along feature dimensions.


$$\text{attention}\left(X\right)=W_{p}•\text{attend}{\left(X,A^{\left(i\right)}\right)}_{i\in \left\{1,\cdots ,n_{h}\right\}}$$

Where, the *A* can be the separate attention patterns, the merged patterns.

### 3.7 D-SA-FCN network

#### 3.7.1 FCN network

FCN classifies images at pixel level, thus solving the problem of image segmentation at semantic level. Different from the classic CNN, which uses the fully connected layer after the convolutional layer to obtain feature vectors of fixed length for classification, FCN can accept input images of arbitrary size. The deconvolution layer is used to up sample the feature map of the last convolutional layer to restore it to the same size as the input image. Thus, a prediction is generated for each pixel, and the spatial information in the original input image is preserved. Finally, pixel-by-pixel classification is performed on the up sampled feature map.

#### 3.7.2 Dual FCN network

The input diversity of this paper includes an array of optimized eigenvalues and a matrix array of processed microscopic images, and there is a large difference between the two groups of inputs. Therefore, a Dual FCN neural network is designed, and its mathematical model is as follows:


$$\{\begin{array}{c} \begin{array}{c} F\left[t\left(x\right)\right]=F^{\left(N\right)}\left\{F^{\left(N-1\right)}\cdots F^{\left(1\right)}\left[t\left(x\right)\right]\right\}\;\\ t\left(x\right)=s\left[f\left(x\right),g\left(x\right)\right] \end{array}\\ \begin{array}{c} f\left(x\right)=\;f^{\left(N\right)}f^{\left(N-1\right)}\cdots f^{\left(1\right)}\left(x\right)\;\\ g\left(x\right)=\;g^{\left(N\right)}g^{\left(N-1\right)}\cdots g^{\left(1\right)}\left(x\right)\; \end{array} \end{array}$$

Where ***f*** (*x*) represents the multi-layer perceptron with the optimized array of eigenvalues as the input; ***g*(*x*)** represents the multilayer perceptron with a matrix array of processed microscopic images as input; ***t*(*x*)** means that ***f*(*x*)** and ***g*(*x*)** are combined and randomly shuffled; ***F*** [***t*(*x*)**] represents a multilayer perceptron with mixed features as input.

#### 3.7.3 D-SA-FCN network

**Figure 8:**
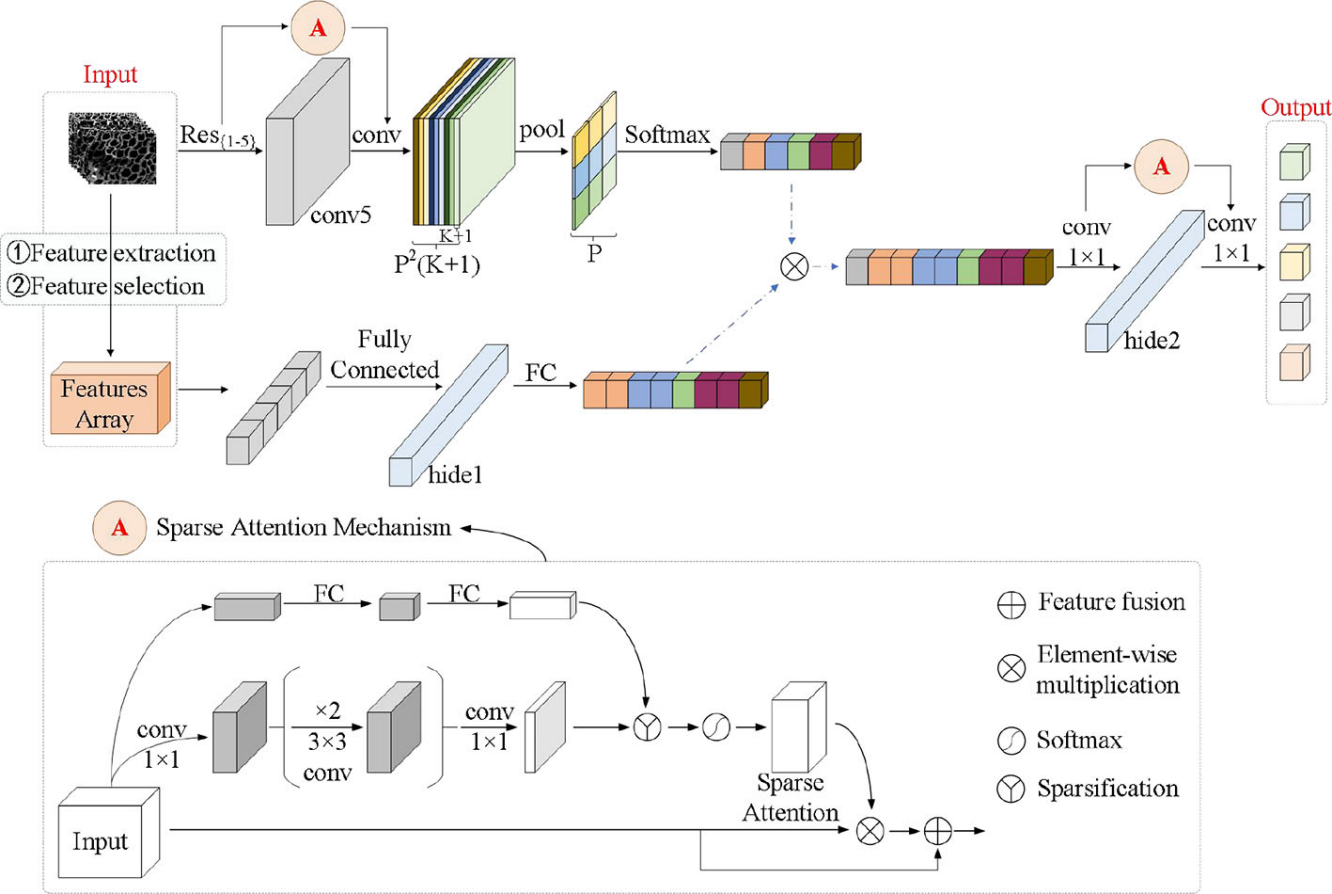
D-SA-FCN network model structure.

As shown in **Figure 8**, compared with the traditional CNN neural network, the FCN neural network replaces the fully connected layer at the back of the network with a 1×1 convolutional layer, so that it can accept input images of any size and realize pixel-level classification. For the FCN neural network with the feature matrix and microscopic image as input, the input layer has great differences. A network model with two inputs is de-signed, and the feature matrix and microscopic image are assumed to undergo different processing layer mechanisms. After extracting the respective classification features, feature fusion is performed to obtain the fusion features, and then the full convolution model is used to complete the final classification prediction.

In this paper, the realization of the classification model mainly includes initialization and model training. Initialization includes setting neural network parameters, building network structure, setting loss function and setting optimizer. According to the empirical value, the learning rate is set to 0.01, with a total of 500 batches, each batch size is 128. The sigmoid function is selected for the input layer activation function, and the element value is mapped between [0,1], which is beneficial to accelerate the network learning speed. The Sigmoid function is defined as:


Sigmoid(x)=1/[1+exp(−x)]

For the activation function of the hidden layer, ELU function is selected to accelerate the convergence speed. The ELU function is defined as:


$$ELU\left(x\right)=\{\begin{array}{c} x\\ \alpha \left(e^{x}-1\right) \end{array}\;\;\begin{array}{c} x\geq 0\\ x<0 \end{array}$$

Because of the multi-classification model in this paper, Softmax function is selected as the activation function of the output layer. The Softmax function is defined as:


$$Softmax\left(x_{i}\right)=e^{x_{i}}/\sum_{c=1}^{C}e^{x_{c}}$$

Where, *x*_*i*_ is the output value of the *i* th node, and *C* is the number of output nodes, that is, the number of categories of classification.

The loss function selects cross entropy, and its multi-classification formula is defined as:


$$L=\frac{1}{N}\sum_{i}L_{i}=\frac{1}{N}\sum_{i}\sum_{c=1}^{M}y_{ic}log\left(p_{ic}\right)$$

Where *M* is the number of categories, *y*_*ic*_ is the sign function (if the true category of sample *i* is *c* take 1, otherwise take 0), *p*_*ic*_ is the predicted probability that the observed sample *i* belongs to category *c* .

The optimizer selects the stochastic gradient optimization algorithm SGD.

The model accuracy is different from the conventional random forest model accuracy algorithm, and the formula is as follows:


$$Accuracy=\left(T_{1}+T_{2}+T_{3}+T_{4}+T_{5}\right)/T_{ALL}$$

Where, *T*_1_ , *T*_2_ , *T*_3_ , *T*_4_ and *T*_5_ are the number of correct samples as Ic, IIc, IIIc, IVc and Vc, respectively; *T*_*ALL*_ is the number of all samples.

## 4 Result and discussion

### 4.1 Model results

**Figure 9:**
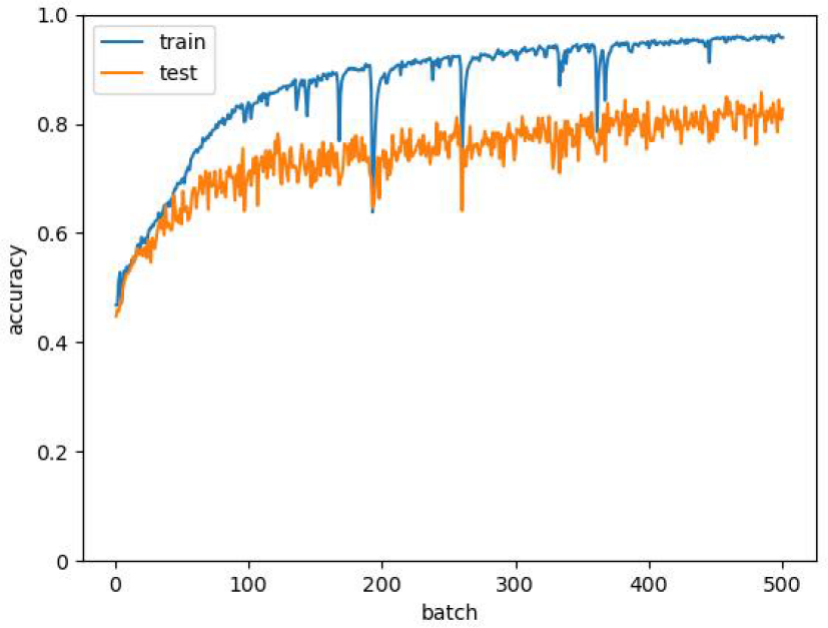
Accuracy of D-SA-FCN model training set and test set.

The neural network was set to 500 batches, the size of each batch was 128, and the model learning rate was 0.01. The samples were divided into training sets and test sets in a ratio of 8:2. The D-SA-FCN network training effect is shown in **Figure 9**. The accuracy of the final training is 96.87%, and that of the test is 85.75%.

**Figure 10:**
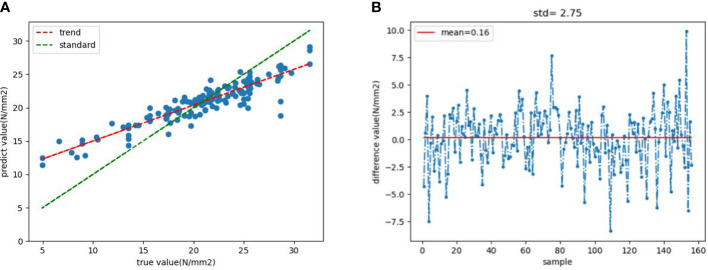
**(A)** True value and predicted value comparison chart and trend line **(B)** The margin of true value and predicted value.

**Figure 10A** shows the comparison between the real value and the predicted value of the test set. The predicted trend line and standard line are drawn according to the predicted situation. It can be seen that the prediction of the intermediate value is closer to the real value. **Figure 10B** shows the margin of the real data value and the predicted value. The mean and standard deviation of the calculated travel value are 0.16 and 2.75, indicating that the prediction is relatively accurate.

**Figure 11:**
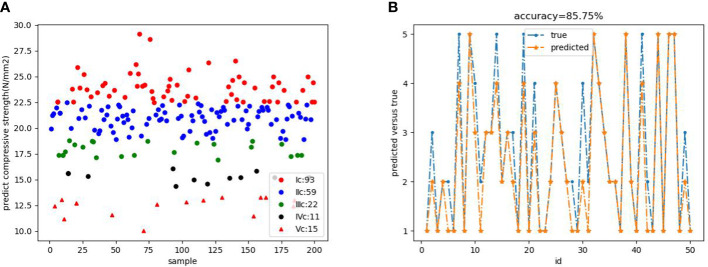
**(A)** Predicted classification result map **(B)** Comparison between test set classification results and actual results.

According to the established classification basis, complete the classification of the predicted value of the model. **Figure 11A** shows the result of the prediction classification. The test set classification level and actual level are shown in **Figure 11B**. By comparing and analyzing the predicted classification and the real classification of the test set, the ac-curacy of the model was calculated according to the formula, and the final accuracy was 85.75%.

### 4.2 Data analysis

The comparison results between the accuracy of the D-SA-FCN training model and the accuracy of the random forest and regression models are shown in **Table 6**. It can be seen that the influence of each eigenvalue on mechanical properties is from large to small, which are CON, CORR, AREA, PERIMETER and ASM. Furthermore, the feature array with texture features and morphological features as inputs was input into the D-SA-FCN to train the model. The accuracy rates were 79.52% and 71.48%, respectively. By comparison, it is found that the tracheid texture features of the larch microscopic image have a greater effect on its mechanical properties.

**Table 6:** Features and mechanical properties response.

Feature	CON	CORR	AREA	PERIMETER	ASM
Random forest	23%	21%	20%	18%	18%
Regression algorithm	24.81%	22.43%	17.28%	15.20%	14.02%
**D-SA-FCN**	**66.32%**	**62.53%**	**59.78%**	**53.25%**	**47.83%**

The bold part indicates the method used in this paper.

### 4.3 Ablation experiment

In order to verify the effect of model optimization, ablation experiments were conducted on network channels, attention mechanism and data enhancement in the experiment, and the experimental results are shown in **Table 7**. The results show that using dual channels instead of single channels, increasing attention mechanism and data enhancement can improve the accuracy of the model.

**Table 7:** Ablation Experiment.

Channel	Attention	Data enhance	Accuracy
single	**/**	**/**	73.37%
single	**/**	√	78.36%
double	**/**	√	80.32%
double	Spatial	√	83.26%
double	Atrous	√	83.43%
double	Local	√	83.71%
**double**	**Sparse**	**√**	**85.75%**

The bold part indicates the method used in this paper.

### 4.4 Comparison

As shown in **Table 8**, the D-SA-FCN network model is superior to the random forest model, CNN network model, Residual network model, single FCN network model, double FCN network model, and Bayesian network model in terms of performance. The accuracy of the test set is improved by 16.5%, 10.22%, 8.71%, 7.39%, 5.24%, and 4.19% respectively. Compared with the classical machine learning model random forest model, the model in this paper adopts deep learning algorithm, introduces feedforward and recursive network, which greatly im-proves the accuracy of prediction. Compared with CNN network and Residual network, the model in this paper changes the final fully connected layer to the convolution layer with specific parameters, and changes the classification level from image to pixel level. The single FCN model for all input used the same way, lack of correlation between the input parameters of digging. Although the Bayesian network model can fully describe the relationship between data, there are some shortcomings in the weight calculation of different input parameters. The D-SA-FCN network model designed in this paper sets up two different processing mode layers for different input parameters, sets up a fusion mechanism for extracted features, and then further carries out convolution processing to more accurately mine the correlation between each input parameter and between input and output, and further improves the accuracy of the model.

**Table 8:** Model comparison.

Model	Test set accuracy
Random forest	69.25%
CNN network	75.53%
Residual network	77.04%
Single FCN network	78.36%
Double FCN network	80.32%
Bayesian network	81.56%
**D-SA-FCN**	**85.75%**

The bold part indicates the method used in this paper.

## 5 Conclusion

In this scheme, larch in the Greater Khingan Mountains of China was taken as the specimen, the optical scanning microscope microscopic images were taken as the texture features, the gray co-occurrence matrix was taken as the morphological features, such as the area and perimeter of the average tracheid after cell segmentation, and the compression strength parallel to the grain of wood was taken as the mechanical property basis. The traditional FCN network model was improved, and the dual channel mechanism and sparse attention mechanism were introduced to improve the robustness of the model, and the mechanical property neural network classification of D-SA-FCN model was established.

The optimized feature array and simplified microscopic images were used as the input of D-SA-FCN neural network model to complete the five-level classification of larch mechanical properties according to national standards. The accuracy of training set and test set reached 96.87% and 85.75%, respectively. When morphological and texture features are input, the accuracy of the training model is 71.48% and 79.52%, respectively. Texture feature has a more significant effect on mechanics to a certain extent. The D-SA-FCN network model can reduce the computational complexity, improve the prediction accuracy and meet the requirements of wood micromechanical properties research by completing ablation experiments on the direction of channel, attention and data enhancement.

This paper also needs to be improved in the following aspects. First, there are only two kinds of tracheid morphological feature extracted, so parameters such as tracheid microfiber Angle and spectral features can be attempted. Second, five classifications of mechanical properties have been completed according to the national standards, and the number of classifications is small. We can try to carry out more detailed classification on the basis of the national standards. Thirdly, the mechanical properties of wood are only classified based on the compression strength parallel to the grain. The flexural strength parallel to the grain and the horizontal grain can be added as the mechanical property indexes to study the correlation between the micro and macro properties of wood.

## Data availability statement

The raw data supporting the conclusions of this article will be made available by the authors, without undue reservation.

## Author contributions

Conceptualization, CL and LZ. Resources, XC. Data curation, SW. Writing—original draft preparation, LZ. Writing—review and editing, CL. Supervision, WJ. All authors have read and agreed to the published version of the manuscript.
